# Varieties of perceptual instability and their neural correlates

**DOI:** 10.1016/j.neuroimage.2014.01.040

**Published:** 2014-05-01

**Authors:** Tomohiro Ishizu, Semir Zeki

**Affiliations:** Wellcome Laboratory of Neurobiology and Wellcome Department of Imaging Neuroscience, University College London, Gower Street, London WC1E 6BT, United Kingdom

**Keywords:** Perceptual reversal, Cross categorical boundary, Anterior cingulate cortex, Ambiguity, Conflict resolution

## Abstract

We report experiments designed to learn whether different kinds of perceptually unstable visual images engage different neural mechanisms. 21 subjects viewed two types of bi-stable images while we scanned the activity in their brains with functional magnetic resonance imaging (fMRI); in one (intra-categorical type) the two percepts remained within the same category (e.g. face–face) while in the other (cross-categorical type) they crossed categorical boundaries (e.g. face–body). The results showed that cross- and intra-categorical reversals share a common reversal-related neural circuitry, which includes fronto-parietal cortex and primary visual cortex (area V1). Cross-categorical reversals alone engaged additional areas, notably anterior cingulate cortex and superior temporal gyrus, which have been posited to be involved in conflict resolution.

## Highlights

• fMRI reveals brain mechanisms involved in viewing different types of unstable stimuli.
• Fronto-parietal cortex and V1 are activated by all visually unstable stimuli.
• Perception of different categories in unstable stimuli activates ACC and STG.
• Studies of unstable stimuli give insights into how brain resolves sensory conflicts.

## Introduction

In trying to obtain knowledge about the external world, the brain employs two strategies; one consists of stabilizing the world by discarding all the superfluous and inconstant changes, one example of which is the generation of constant colours, which makes the brain independent of the continual changes in wavelength-energy composition of the light reflected from objects and surfaces (Land, 1974; Zeki, 1980), thus giving us knowledge about them through their colour. But there are also stimuli whose configuration is such that they are sources of uncertain knowledge since they remain open to more than one interpretation, classic examples being the Necker Cube and the Rubin Vase. To allow for the uncertain knowledge derived from such perceptually unstable stimuli, the brain appears to have evolved another strategy — a system that is stable in its instability, in the sense that it can allow for more than one interpretation of a stimulus even when higher cognitive factors impel the interpretation in one direction (Zeki, 2008). This phenomenon has been especially useful in investigating neural systems that correlate with the conscious perception of objects and previous neuroimaging studies have shown that frontal and parietal cortex and functionally specialized visual areas are engaged in all perceptual reversals (e.g. Andrews and Schluppeck, 2004; Kanai et al., 2010; Kleinschmidt et al., 1998; Lumer et al., 1998).

Bi-stable figures can be divided broadly into two sub-types, those showing *intra-categorical* and those showing *cross-categorical* reversals. The Necker cube belongs to the former: it is perceived as one object (category) that can be in one of two recessional states. The Rubin vase belongs to the cross-categorical variety, in that it can alternate perceptually between two categories (face and vase). In both, there is an *obligate bi-stability* (and an obligate multi-stability in others) since it is difficult, if not impossible, to prevent the transition from one state to the other; both are perceptually “correct” at any given moment and each occupies sovereignly the conscious perceptual stage for a given moment before the other interpretation replaces it to supervene. This makes it especially interesting to compare the brain activity produced by the two types of bi-stability. The cross-categorical transition results in a conflict condition since the perceptual transition is between two different categories; by contrast, the transition in intra-categorical examples is only a change from one state to another of the same category (object) and hence introduces no categorical conflict in what the image is, but only in its configuration. In this study, we therefore wanted to learn whether the same neural circuits are engaged in the two kinds of bi-stability. Our working hypothesis was that intra-categorical and cross-categorical bi-stability will both engage the same fronto-parietal areas but that the cross-categorical bi-stability will engage cortical areas that have been shown to be prominently engaged in conflict resolution, principally the anterior cingulate cortex (ACC). We hypothesized further that activity in the ACC would be restricted to the reversal phase of the unstable images, since it is then alone that a conflict arises about the knowledge derived from the stimulus. Hence our study was specifically focused on the engagement of the ACC in the two kinds of obligate bi-stability, to learn whether activity in it would allow us to distinguish neurologically between the two kinds of perceptual transition.

To do so, we used functional magnetic resonance imaging (fMRI) to scan brain activity when subjects viewed both types of bi-stable figures and indicated when their percepts alternated between two interpretations; this allowed us to distinguish between activity that correlates with the reported perceptual reversal for both types of stimuli.

## Materials and methods

### Subjects

21 healthy right-handed volunteers (9 male, 12 female, mean age 23.7 years) participated. All had normal or corrected-to-normal vision, and none had a history of neurological or psychiatric disorder. Written informed consent was obtained from all. The study was approved by the Ethics Committee of University College London and conforms with the Code of Ethics of the World Medical Association (Declaration of Helsinki; printed in the British Medical Journal 18 July 1964). All data was anonymized.

### Procedure

Subjects were instructed to report repetitively their perception of visual stimuli during stimulus presentation and to press one of two buttons with their right hand to indicate one or the other of the two possible directions of perceptual transitions. After reporting a transition, they kept the key pressed while the ensuing percept was stable. We calculated middle-points for each time-interval between each button press and release (that is, during the period when subjects' perception was stable) and used these as the onsets for stable perception in an event-related analysis (Kleinschmidt et al., 1998). Subjects' reports thus defined the occurrence of perceptual reversals and the presence of stable perceptions. There were, therefore, two critical time-points, when a reversal occurred and when perception was stable.

**Fig. 1:**
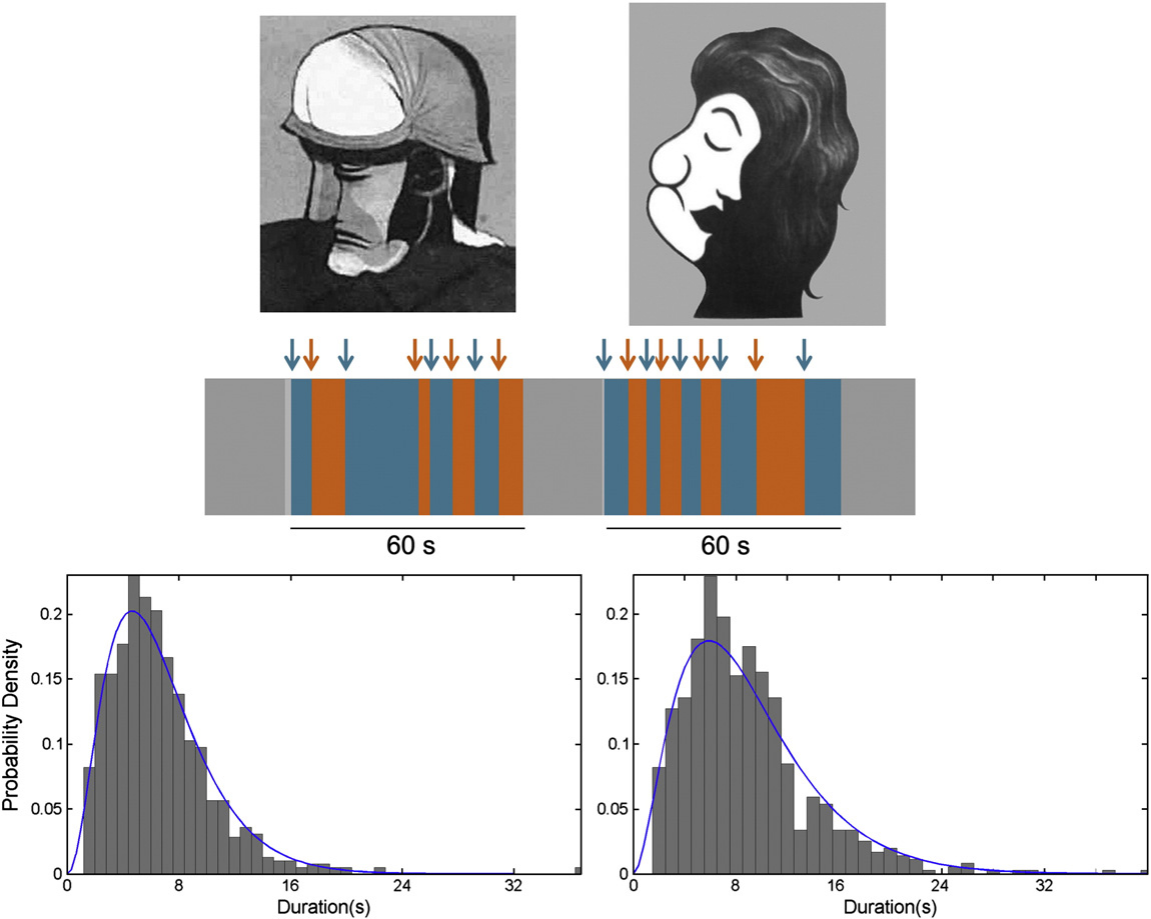
Upper panel: examples of bi-stable figures used in the experiment. To the left, a *face*–*body* bi-stabile figure is an example of cross-categorical reversal, and to the right, a *face*–*face* bi-stabile figure is one for intra-categorical reversal. Middle panel: illustrative example of responses while viewing a bi-stable stimulus, derived from one subject's data. Blue phases correspond to one percept and orange ones to another. Lower panel: the distributions of grand averaged inter-reversal times (durations of percepts) reported by participants. The blue lines show the fitted gamma distribution.

We used a variety of bi-stable figures belonging to two classes of stimuli: in one class (cross-categorical) were stimuli for which perception alternates between one category and another (e.g. between a face and a body or a vase and faces, see Fig. 1) while in the other (intra-categorical) class were stimuli in which the transition is between two percepts belonging to the same category (e.g. from one face to another, see Fig. 1). Table 1 provides all stimulus categories used in the experiment. Preceding the fMRI experiments, participants viewed all bi-stable figures and familiarised themselves with the stimuli. At the beginning of each trial in the scanning sessions, subjects were informed what stimulus categories would appear in the coming trial, with an instruction for which button to press to indicate their percepts (e.g. “*Face = left button, Body = right button*”). The stimuli covered approximately 8° × 8° of the visual field and included a central fixation cross. Inter-reversal time-spans can be controlled with parameters that depend on stimulus type, visual field coverage and fixation point (Borsellino et al., 1982; Kleinschmidt et al., 1998). These stimulus settings followed a previous study using bi-stable figures (Kleinschmidt et al., 1998). All stimuli were grey-scaled pictures and the brightness of each was adjusted to the averaged brightness of all. Stimulus presentation lasted for 60 s followed by a 20 s fixation period during which the fixation cross was presented on a black background. Subjects were instructed to fixate the cross throughout scanning. There were 8 stimuli for each reversal condition, resulting in 16 experimental stimuli in total. The experiment consisted of 4 scanning sessions and each session had 4 stimuli. The order of the stimulus presentation and button-pressing were counterbalanced across subjects. Number of reversals and durations of percepts between intra- and cross-categorical conditions were analysed using *t*-tests.

### Functional image acquisition and analysis

Scanning data was acquired in a 3-T Siemens Magnetom Trio MRI scanner (Siemens, Erlangen, Germany) fitted with a 12-channel head-coil. An echo-planar imaging sequence was applied for functional scans to obtain blood oxygen level dependent (BOLD) signal (TR = 68 ms, TE = 30 ms, matrix size = 64 × 64) using 48 slices to cover the whole brain. The voxel resolution was 3 × 3 mm in-plane resolution, with a 2 mm slice thickness and 1 mm inter-slice gap. High-resolution T1-weighted anatomical images were acquired at the end of experimental sessions for each subject (176 slices, resolution 1 × 1 × 1 mm, TE = 2.48 ms, TR = 7.92 ms). Field maps were also acquired with Siemens standard gradient-echo field map sequence for correcting geometric distortion of echo-planar imaging (EPI) images (Hutton et al., 2002). We also recorded the heart and respiration rates for each subject.

All data were analysed using SPM8 (Statistical Parametric Mapping http://www.fil.ion.ucl.ac.uk/spm/software/spm8/). The EPI images for each subject were realigned and normalized into Montreal Neurological Institute (MNI) space, smoothed using Gaussian smoothing kernel of 9 × 9 × 9 mm, and filtered with a high-pass cutoff (128 s) to remove drift terms. The stimulus for each subject was modelled as a set of regressors in a general linear model (GLM) first-level (within subject) analysis.

The study used an event-related design which models the evoked haemodynamic responses for events (key-presses indicating perceptual reversal and calculated onsets indicating stable perception) as delta functions convolved with a canonical haemodynamic response function with time and dispersion derivatives to provide regressors for the GLM. Head movement parameters calculated from the realignment pre-processing step and physiological recordings (heart rate, respiration) were included as regressors of no interest. We carried out categorical contrast analyses encoding the same data in two ways. For the first we used separate stimulus functions for *reversal* and *stable* events and pooled all events regardless of category, while for the second categorical analysis we used separate stimulus functions for cross-categorical and intra-categorical *reversal* and *stable* events. Contrast images were taken to second-level (between subject) *t*-tests to produce summary t-statistical maps at the group level.

We report cluster level activations significant at *p* < 0.05 family wise error (FWE) corrected, although some of these (indicated in the tables) were significant at peak level at *p* < 0.05 FWE corrected. In cases where we had *a priori* knowledge of an area's involvement, we used a small volume correction (SVC) of 16 mm, *p* < 0.05 corrected at voxel level, using coordinates given in previous studies (Kanai et al., 2008; Knapen et al., 2011).

In post-hoc sessions, eye-movements were measured in six subjects by using an eyetracker (Eyelink system, SR Research, Berlin, Germany) in the fMRI scanner. The eye-movement data was calibrated with nine points and sampled at 250 Hz. The data were classified according to the two reversal conditions.

## Results

### Behavioural data

All subjects reported frequent perceptual reversals when viewing the stimuli. Inter-reversal times for each condition, while individually variable, are strongly shifted toward the left and can be approximated by gamma distributions shown in Fig. 1, which is a typical feature of perceptual reversal phenomena (see Taylor and Aldridge, 1974; Leopold and Logothetis, 1999 for reviews). There was no significant difference between intra- and cross-categorical conditions in the number of reversals (intra-categorical, mean = 9.1, cross-categorical, mean = 9.81, *p* > 0.05) and the durations of percepts (intra-categorical, mean = 7.4 s, cross-categorical, mean = 6.8 s, *p* > 0.05).

### fMRI data

Our specific aim was to learn whether, in addition to the fronto-parietal cortex, which appears to be engaged during all types of perceptual reversal, the ACC is differentially involved in the two types of reversal. We used (1) the contrast *perceptual reversal* > *perceptual stability* to chart general brain activations that correlate with perceptual switching and (2) the contrast *cross-categorical perceptual reversals* > *intra-categorical perceptual reversals* to chart brain regions that are especially active in cross-categorical perceptual reversals. We also used a conjunction analysis (Price and Friston, 1997) to characterize brain activations common to both types of perceptual reversal using the contrast [*cross-categorical reversal* > *cross-categorical stability*] and [*intra-categorical reversal* > *intra-categorical stability*]. We also report activations for each of the following contrasts; *cross-categorical reversal* > *cross-categorical stability* and *intra-categorical reversal* > *intra-categorical stability*. Activations for all contrasts are summarized in Table 2.

### Perceptual reversal vs perceptual stability

**Fig. 2:**
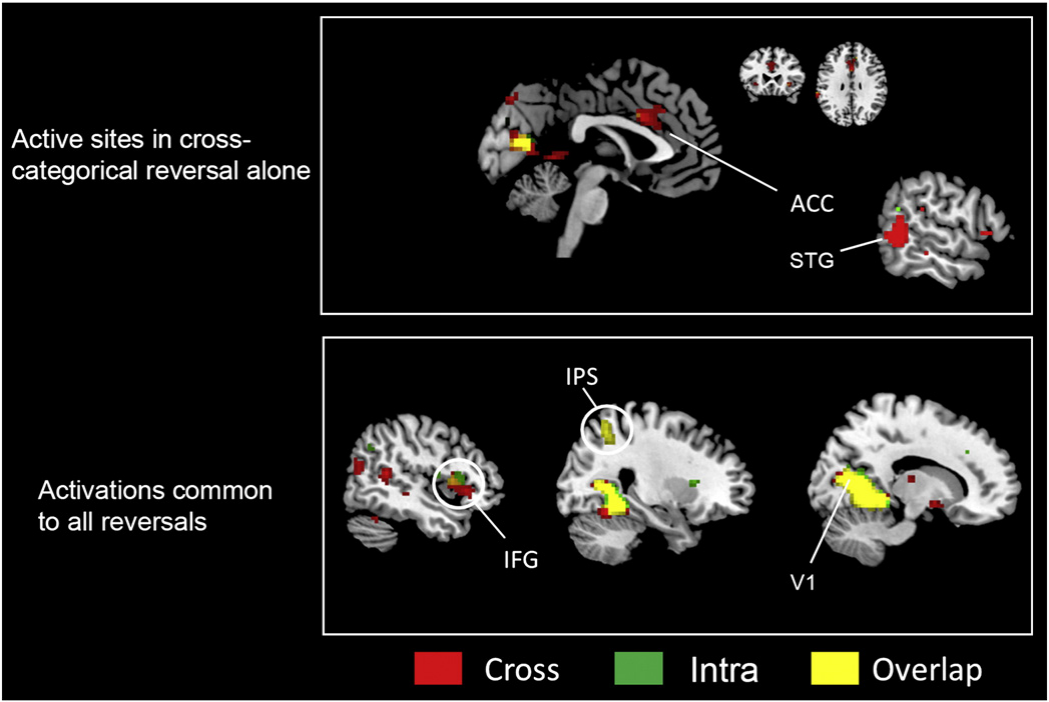
Upper panel shows sites that were active during cross-categorical reversals and lower panel shows ones active during both cross- and intra-categorical reversals. Statistical parametric maps rendered onto canonical anatomical sections showing *t*-statistics. Random effects analysis with 21 subjects. Display threshold *p* < 0.001 (uncorrected). ACC, anterior cingulate cortex (0 17 28); STG, superior temporal gyrus (60 − 49 1); IFG, inferior frontal gyrus (51 23 − 5); IPS, intraparietal sulcus (21 − 49 43); V1, primary visual cortex (− 12 − 73 10).

(a) The contrast *perceptual reversal* > *perceptual stability* for both categories of stimulus led to activation in bilateral inferior frontal gyrus (IFG) and right intraparietal sulcus (IPS) (Fig. 2), which have been reported to be active in previous studies using ambiguous (intra-categorical) figures like the Necker cube (e.g. Pitts et al., 2007) and bi-stable stimuli (Kleinschmidt et al., 1998; Leopold and Logothetis, 1999; Lumer et al., 1998, *inter alia*). These fronto-parietal activations may reflect top-down processes that initiate a reorganization of activity throughout the visual cortex during perceptual reversals (Leopold and Logothetis, 1999). Activation in the primary visual cortex (V1) (encroaching upon cuneus and parahippocampal gyrus), previously reported for figure-ground reversal (Polonsky et al., 2000) and during binocular rivalry (Parkkonen et al., 2008), was also found. In addition, there was activation in bilateral anterior insula and the ACC, areas which, together with the front-parietal cortex, are thought to play a role in visual awareness (Rees, 2007).

(b) The contrast *perceptual stability* > *perceptual reversal* for both categories of stimulus led to activity in the following areas: occipital and parietal cortex, including lingual gyrus (probably area V3) and middle occipital gyrus bilaterally, the supplementary motor area and paracentral area. The role of V1 in perceptual reversal is controversial (de-Wit et al., 2012); there are contradictory suggestions as to whether it is engaged during perceptual reversal (e.g. Polonsky et al., 2000) or not (e.g. Kleinschmidt et al., 1998) (see also discussion). In this study, activations in visual cortex with perceptual stability were located ventrolaterally (24−88 − 5; − 24 − 88 − 8) outside V1, while those induced by perceptual reversal were in V1 (18 − 67 7; 24 − 49 − 8). Kleinschmidt et al. (1998) reported activations with perceptual stability at similar coordinates, whereas the locations of activation reported in the studies of Polonsky et al. (2000) and Parkkonen et al. (2008) are located in V1 and thus posterior relative to our activation foci.

Other activations are summarised in Table 2.

### Cross- and intra-categorical conditions

Our main interest was to learn whether there are any significant differences in the pattern of cortical activation produced by cross-categorical reversals as opposed to intra-categorical ones and in particular whether the ACC is involved in one but not the other.

*Areas uniquely active in each kind of reversal*: the significant difference in activation between the two types of reversal may be summarized as follows: ACC and right superior temporal gyrus (STG) were engaged during cross-categorical but not intra-categorical reversal (Fig. 2). In addition to its involvement in visual awareness (Rees, 2007), the ACC is also engaged in a wide range of cognitive tasks (Botvinick et al., 2001; 2004 for reviews), including ones which involve conflict (e.g. Botvinick et al., 2001; Carter et al., 1995; Raichle et al., 1994). Previous neuroimaging studies using Stroop tasks or audio dichoptic competition have suggested that the STG is also involved in conflict resolution (e.g. Falkenberg et al., 2011; Pardo et al., 1990). By contrast, there was no comparable unique activation in the intra-categorical reversal contrast, which shared all activations with the cross-categorical one at the significance level (see common activations below). It should be noted that there was no activity in the ACC and STG with both types of stability.

*Areas commonly active in both kinds of reversal*: there were also significant similarities in the activation between the two types of reversal, which may be summarised as follows: IFG and IPS (fronto-parietal area) and V1 (Fig. 2). The latter was previously reported to be active during the viewing of multi-stable reversal stimuli (e.g. Tong et al., 2006) (see also discussion).

Eye-movement tracking data revealed indistinguishable gaze patterns between the two reversal conditions, suggesting that the differences in the activation patterns produced by the two reversal conditions cannot be accounted for by eye-movement patterns during the perceptual reversals.

## Discussion

For the purposes of the present study, we have divided ambiguous stimuli into two broad types, those in which the reversals do not cross categorical boundaries and those that do, to learn the extent to which the two engage different neural mechanisms. Past studies have shown that reversals of both kinds engage the fronto-parietal cortex but, given their profound perceptual difference, it seemed reasonable to expect that, beyond the basic neural mechanisms engaged in reversals in general, there might be additional differences related to the type of reversal. More specifically, the crossing of categorical boundaries introduces a conflict as to which category a stimulus belongs to – a conflict that is, by its nature, only temporarily resolved perceptually – while an intra-categorical transition does not produce a conflict between perceptual categories but only between different states or configurations of the same category of object. We thus focussed our study on the possible involvement of the ACC, known from previous studies to be involved in conflict resolution. Collectively, our results show that, as expected, cross- and intra-categorical reversals share a common reversal-related neural circuitry but additional areas, notably the ACC and the STG, are recruited in cross-categorical reversals alone. We begin by discussing the general activation produced by both types of ambiguous stimuli before discussing more specifically the central results.

### Area V1

Although not the major results of this study, the activity in area V1 raises interesting questions: For both intra- and cross-categorical stimuli, we observed activation in V1 with *perceptual reversal* > *perceptual stability* but not for the reverse contrast. It is as if V1 is engaged in the sensory configuration of the visual stimulus but not in the interpretation of the stable percept. Although previous electrophysiological and some early fMRI studies did not report V1 activation with perceptual transitions (e.g. Kleinschmidt et al., 1998; Leopold and Logothetis, 1999; Lumer et al., 1998), many neuroimaging studies using fMRI and magnetoencephalography have suggested that V1 may be engaged in various bi-stable percepts, including binocular rivalry (Polonsky et al., 2000; Tong and Engel, 2001), apparent motion (Seghier et al., 2000) and static illusory contours (Muckli et al., 2009). This inconsistency may be explained by general differences in methodologies (Sterzer et al., 2009). It is known that neuronal spiking activity is less closely related to perceptual awareness than local field potentials (Wilke et al., 2006), which correlates more with the BOLD signal (Logothetis et al., 2001). The current results support the involvement of V1 in perceptual reversal but not in perceptual stability, by showing that V1 is co-activated with fronto-parietal cortex during perceptual reversals alone. However, the causal and temporal relationship between V1 and fronto-parietal cortex during perceptual reversal is still unclear. Transcranial magnetic stimulation and fMRI connectivity analysis (Kanai et al., 2010; Sterzer and Kleinschmidt, 2007) suggest that fronto-parietal cortex may play a crucial role in perceptual reversal by sending signals to sensory areas but whether activity in fronto-parietal cortex is antecedent to V1 activity or subsequent to it remains unclear.

### ACC, STG and cross-categorical reversals

**Fig. 3:**
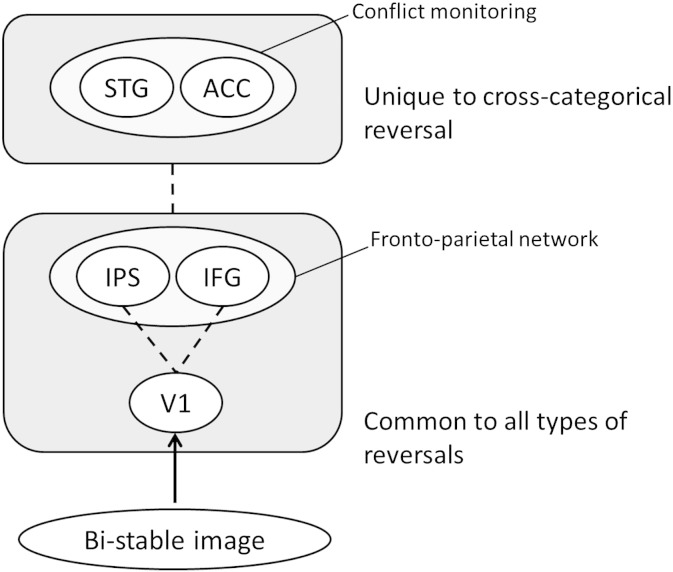
Summary diagram. A proposed hypothetical scheme to illustrate brain mechanisms underlying perceptual reversal. Lower panel shows brain areas involved commonly in all types of reversals, cross- and intra-categorical (V1, IPS, and IFG). Upper panel shows areas involved in cross-categorical reversal alone (STG and ACC). The temporal relationship between the two mechanisms is unclear.

Returning to the main aims of this study, we found, as we had hypothesised, that activity within the ACC is restricted to cross-categorical reversals alone, not during perceptually stable states. Our measurements confirmed that this activity cannot be accounted for by eye-movements. Hence the activity must be limited in duration to conditions in which neither percept supervenes, resulting in the creation of a perceptual conflict. In light of this, we suggest that the activation of ACC (and STG) reflects a conflict in matching between two possible perceptual interpretations of the stimulus. The only previous fMRI study of the neural correlates of perceptual reversal in complex bi-stable figures (Kleinschmidt et al., 1998) did not aim to distinguish between cross- and intra-categorical reversals and therefore did not report activation in the ACC. Perceptual reversal is not unique to the visual modality; it also occurs in the auditory one (e.g. Kashino and Kondo, 2012) and the two may share, at least partially, a common neural circuitry during perceptual reversal (Kondo et al., 2012; Sterzer et al., 2009). Both, for example, engage the sensory areas (auditory cortex in the case of auditory stimuli and visual cortex for visual stimuli) and parietal cortex (Sterzer et al., 2009; Kashino and Kondo, 2012 for a review). They also apparently share the common feature that, where the bi-stability crosses categorical boundaries, the ACC is involved, since with auditory instability the ACC is only engaged with so-called verbal transformation effects (Sato et al., 2004). For example, when a word such as “life” is repeated rapidly and continuously listeners' perception of the word will alter between ‘life’ and ‘fly’ (e.g. Warren and Gregory, 1958). There may in fact be further analogies between them. A tone is a tone and a face is a face, but ‘life’ is not ‘fly’ and a ‘face’ is not a ‘vase’; hence they belong to different ‘conceptual’ categories within the two sensory domains. We therefore propose that, in addition to the activation of general reversal-related neural circuits, the ACC comes into play when the two interpretations belong to different conceptual categories and lead to conceptual conflict, no matter what the source is (see also Fig. 3 for a summary diagram).

In addition to the ACC, the STG was also only active in cross-categorical instability. The STG is a multi-functional region which is thought to play a role in insight problem solving (Bowden et al., 2005) and attention control (Wojciulik and Kanwisher, 1999); interestingly it has also been reported to be engaged in conflict solving (Falkenberg et al., 2011; Pardo et al., 1990).

### Conflict resolution

The restriction of ACC and STG activity to phases of reversal in cross-categorical transitions raises a number of issues. It is, first, important to note that a change in the stimulus itself does not necessarily trigger a response in these two areas. For example, when the wavelength composition of light reflected from the coloured patches of a Mondrian display changes continually, without leading to a change in perceived colour (though there is a change in hue), the ACC and the STG are not engaged (Bartels and Zeki, 2000). Hence, it appears that these two areas are engaged only when the stimulus is neurally, but not veridically, unstable, and only when it crosses categorical boundaries. Next comes the question of conflict resolution. When we speak of it in this context, we are of course intending that the conflict is only resolved momentarily, for a period of seconds or minutes — when one perceptual state supervenes; for periods in excess of that, the conflict is not resolved, for the reversion from one state to the other is continuous, and subjects *know* that they are viewing an unstable stimulus, even if it is momentarily resolved. Hence it is more appropriate in our context to speak, not of a conflict resolution, but of the presence of a conflict during certain limited time intervals, which are resolved perceptually at others. Our results, which show that the ACC and the STG are only recruited during the unstable, reversal, state and not during the stable one, suggest that activity in these areas is perceptually driven, is ‘short term’ and is not influenced by cognitive knowledge about the instability of the stimuli. Indeed, previous studies have shown that ACC activity is more closely related to detecting conflict in the stimulus, possibly in relation to action selection, than to top-down control (for review, see Botvinick et al., 2004). This raises on the one hand the question of the temporal relationship between the areas that are active in reversals in general (including V1 and fronto-parietal cortex) and those that are active uniquely during cross-categorical reversals (ACC and STG). On the other hand, it also raises the question of the relationship, both temporal and physiological, between activity in ACC and STG and higher cognitive areas that are the repository of knowledge about the nature of the stimulus — that is whether it is a perceptually stable or unstable stimulus and whether it crosses a categorical boundary or not. Put more simply, activity in ACC and STG in this study appears to be perceptually driven and persists even in the knowledge that the stimulus is unstable. This, in turn, raises another interesting question that we intend to pursue, namely the difference in terms of cortical activity between perceptually unstable stimuli of the kind we have used here and perceptually stable but cognitively (or affectively) unstable stimuli. Examples of the latter are many, and can be found in many works of art (of which Vermeer's paintings provide a good example), where a single perceptually stable image can be given different interpretations during one viewing and between one viewing and another. Whether the ACC and the STG are also involved when viewing perceptually stable but cognitively unstable images is an intriguing question for future studies.

### Linguistic conflict in cross-categorical transitions

Cross-categorical reversals inevitably lead to a linguistic conflict (“is it a face or a vase?”) and, correspondingly, we found activity within left IFG (Broca's area) with the application of an SVC in the contrast *cross-categorical reversal* > *cross-categorical stable*, but not in the contrast *intra-categorical reversal* > *intra-categorical stable*. The STG, which was equally uniquely active in cross-categorical transitions, is also involved in lexical access and activity in it reflects readiness to engage semantic activation guided by top-down processes, for example, those associated with the ACC (Jung-Beeman, 2005; Tian et al., 2011). Here again, the question of a temporal relationship between activity in ACC and STG on the one hand and higher areas, which dictate the interpretation to be given to a perceptually stable image, becomes one of much interest.

### Cross-categorical and intra-categorical transitions

Finally, there is the question of why ACC and STG activity should be restricted to cross-categorical transitions. One answer is that cross-categorical transitions result in a categorical conflict whereas the intra-categorical ones do not. If the activity in ACC reflects conflict resolution in the context of action selection (Botvinick et al., 2004 for a review), then it stands to reason that there is a more radical action selection process involved with unstable stimuli that belong to different categories than ones in which the two states belong to the same category of stimulus and, correspondingly, more enhanced activity in ACC and STG with cross-categorical perceptual reversals. Yet these answers raise important issues. Somehow, the activity in ACC and STG must be finely regulated from two sources: lower areas such as V1 or auditory cortex which signal that the conflict is perceptually driven, and higher (cognitive) sources which must be relatively inactive or suppressed, to indicate that the conflict is perceptual and does not lie in a cognitive instability. It is for this reason that this study is only a prelude to the more demanding task of learning if, and how, ACC, STG and possibly other areas are involved during the experience of perceptually stable but cognitively unstable stimuli. Thus, taken together, the present study expands on previous knowledge on the neural basis of bi-stable perception and raises important issues for future ones.

## Figures and Tables

**Table 1:** A breakdown of the stimulus categories used and the reversals experienced. The upper panel shows the number of stimuli in each group (*e.g.* face–face) within the two major categories. Thus there were, for example, 4 trials in which stimuli entailing face–face reversals were presented whereas there were 3 trials during which stimuli leading to face–body reversals were presented. The lower panels show the number of times the participants could report perceiving the different categories. Within the 8 trials, the maximum possible number of trials during which a face was perceived was 4 (intra-categorical). To the right, the maximum number of trials during which a face could be perceived was 6 (face–body and face–object); the maximum number of trials during which an object could be perceived was 5, and so on.

Intra-categorical reversal		Cross-categorical reversal	
Face–face	4	Face–body	3
Body–body	2	Face–object	3
Object–object	2	Body–object	2
Trials in which face(s) was perceived	4	Trials in which face(s) was perceived	6
Trials in which body(s) was perceived	2	Trials in which body(s) was perceived	5
Trials in which object(s) was perceived	2	Trials in which object(s) was perceived	5

**Table 2:** Activated areas correlating with perceptual reversal and stability. Locations, MNI co-ordinates, cluster size and values for the activations produced by the contrasts: *Perceptual reversal* > *perceptual stability*, *Perceptual stability* > *perceptual reversal*, *Cross-categorical reversal* > *intra-categorical reversal*, and (*Cross-categorical reversal* > *cross-categorical stability*) + (*intra-categorical reversal* > *intra-categorical stability*), *Cross-categorical reversal* > *cross-categorical stability*, and *Intra-categorical reversal* > *intra-categorical stability*. All activations are cluster level significant at *p* < 0.05 (corrected), although some of these were also significant at peak level. Where we had a priori knowledge of an area's involvement, we applied a small volume correction (SVC) of 16 mm indicated as SVC.

			Cluster p(FWE-cor)	Cluster k	Cluster p(unc)	Peak p(FWE-cor)	Peak T	Peak Z	Peak p(unc)	x {mm}	y {mm}	z {mm}
*Perceptual reversal* > *perceptual stability*												
Calcarine gyrus		R	0.000	1923	0.000	0.000	9.438	5.762	0.000	18	− 67	7
Lingual gyrus		R				0.002	8.215	5.374	0.000	9	− 58	− 8
Lingual gyrus		R				0.003	8.062	5.321	0.000	24	− 49	− 8
Insula		R	0.000	115	0.000	0.026	6.935	4.895	0.000	30	20	− 11
Insula		R				0.490	5.315	4.148	0.000	36	26	1
Putamen		R				0.760	4.878	3.914	0.000	30	5	10
Superior temporal gyrus		R	0.000	218	0.000	0.051	6.616	4.762	0.000	48	− 46	13
Middle temporal gyrus		R				0.236	5.845	4.413	0.000	60	− 46	10
Anterior cingulate cortex		L	0.000	290	0.000	0.078	6.418	4.676	0.000	− 3	32	22
Anterior cingulate cortex		R				0.118	6.227	4.591	0.000	9	32	22
Inferior frontal gyrus		R	0.002	89	0.000	0.249	5.808	4.395	0.000	48	11	7
Inferior frontal gyrus		L	0.039	48	0.003	0.333	5.608	4.297	0.000	− 33	26	− 2
Intraparietal sulcus		R	0.022	37	0.003	0.011	4.235	3.640	0.000	30	− 61	55

*Perceptual stability* > *perceptual reversal*												
Supplementary motor area		L	0.000	168	0.000	0.002	8.206	5.371	0.000	− 6	2	64
Precentral gyrus		L	0.000	230	0.000	0.004	7.851	5.246	0.000	− 33	− 22	58
Lingual gyrus		R	0.000	359	0.000	0.020	7.065	4.947	0.000	24	− 88	− 5
Middle occipital gyrus		R				0.086	6.370	4.655	0.000	33	− 88	4
Inferior occipital gyrus		L	0.000	410	0.000	0.089	6.355	4.648	0.000	− 24	− 88	− 8
Paracentral lobule		R	0.000	213	0.000	0.176	6.038	4.504	0.000	9	− 25	70
Precentral gyrus		R	0.011	65	0.001	0.528	5.252	4.116	0.000	57	− 1	40

*Cross-categorical reversal* > *intra-categorical reversal*												
Anterior cingulate cortex		–	0.020	81	0.003	0.767	3.895	3.715	0.000	0	17	28
Superior temporal gyrus		R	0.002	138	0.000	0.015	5.347	4.929	0.000	60	− 49	1
Inferior frontal gyrus	SVC	L		48		0.011	4.235	3.640	0.000	− 30	21	− 3

(*Cross-categorical reversal* > *cross-categorical stability*) and (*intra-categorical reversal* > *intra-categorical stability*)												
Inferior frontal gyrus		R	0.032	72	0.004	0.421	4.238	4.013	0.000	51	23	− 5
Intraparietal sulcus		R	0.003	22	0.081	0.002	4.110	3.910	0.000	21	− 49	43
Lingual gyrus		R	0.000	1290	0.000	0.003	5.797	5.281	0.000	21	− 52	− 5
Lingual gyrus		L				0.005	5.643	5.161	0.000	− 21	− 55	1
Calcarine gyrus		L				0.009	5.476	5.031	0.000	− 12	− 73	10

*Cross-categorical reversal* > *cross-categorical stability*												
Lingual gyrus		R	0.000	1290	0.000	0.003	5.797	5.281	0.000	21	− 52	− 5
Lingual gyrus		L				0.005	5.643	5.161	0.000	− 21	− 55	1
Calcarine gyrus		L				0.009	5.476	5.031	0.000	− 12	− 73	10
Superior temporal gyrus		R	0.002	138	0.000	0.015	5.347	4.929	0.000	60	− 49	1
Inferior frontal gyrus		R	0.032	72	0.004	0.421	4.238	4.013	0.000	51	23	− 5
Anterior cingulate cortex			0.020	81	0.003	0.767	3.895	3.715	0.000	0	17	28
Intraparietal sulcus	SVC	R	0.003	22	0.081	0.002	4.110	3.910	0.000	21	− 49	43

*Intra-categorical reversal* > *intra-categorical stability*												
Lingual gyrus		R	0.000	1159	0.000	0.000	6.510	5.810	0.000	21	− 52	− 8
Lingual gyrus		L				0.000	6.380	5.720	0.000	− 18	− 49	− 5
Lingual gyrus		L				0.004	5.700	5.210	0.000	− 24	− 58	− 2
Inferior frontal gyrus		R	0.022	55	0.004	0.611	4.238	4.013	0.000	47	22	− 9
Intraparietal sulcus	SVC	R	0.004	17	0.121	0.011	3.600	3.450	0.000	24	− 58	58
